# Metabolomic profiling identifies biomarkers and metabolic impacts of surgery for colorectal cancer

**DOI:** 10.3389/fsurg.2022.913967

**Published:** 2022-08-25

**Authors:** Feng Zhuang, Xuesong Bai, Yang Shi, Le Chang, Wanchao Ai, Juan Du, Wei Liu, Humin Liu, Xukun Zhou, Zhong Wang, Tao Hong

**Affiliations:** ^1^ General Surgery Department, Hospital of Xinjiang Production and Construction Corps, Urumchi, China; ^2^ General Surgery Department, Peking Union Medical College Hospital, China Academy of Medical Science & Peking Union Medical College, Beijing, China

**Keywords:** colorectal cancer, metabolomic profiling, tumorectomy, gamma-linolenic acid (GLA), proline, ascorbate, phenylalanine

## Abstract

**Background:**

Colorectal cancer (CRC) is one of the most common malignant tumors with recurrence and metastasis after surgical resection. This study aimed to identify the physiological changes after surgery and explore metabolites and metabolic pathways with potential prognostic value for CRC.

**Methods:**

An ultra-high-performance liquid chromatography Q-exactive mass spectrometry was used to profile serum metabolites from 67 CRC patients and 50 healthy volunteers. Principal component analysis (PCA) and orthogonal projections to latent structures-discriminant analysis were used to distinguish the internal characteristics of data in different groups. Multivariate statistics were compiled to screen the significant metabolites and metabolic pathways.

**Result:**

A total of 180 metabolites were detected. Under the conditions of variable importance in projection >1 and *p*-value <0.05, 46 differentially expressed metabolites were screened for further pathway enrichment analysis. Based on the Kyoto Encyclopedia of Genes and Genomes database and Small Molecule Pathway Database, three metabolic pathways—arginine and proline metabolism, ascorbate and aldarate metabolism, and phenylalanine metabolism—were significantly altered after surgical resection and identified as associated with the removal of CRC. Notably, gamma-linolenic acid was upregulated in the CRC preoperative patients compared with those in healthy volunteers but returned to healthy levels after surgery.

**Conclusion:**

Through serum-based metabolomics, our study demonstrated the differential metabolic characteristics in CRC patients after surgery compared with those before surgery. Our results suggested that metabonomic analysis may be a powerful method for exploring physiological alterations in CRC patients after surgery as well as a useful tool for identifying candidate biomarkers and monitoring disease recurrence.

## Introduction

Colorectal cancer (CRC) ranks third among malignant tumors and second among the leading causes of cancer-related deaths worldwide (1). Despite the great advances in early detection and multimodality therapy for CRC (2), the outcome of patients with advanced tumors is still not satisfactory. Distant metastasis and recurrence are the major causes of death in patients with CRC (3). Therefore, there is an urgent need to better understand the underlying mechanisms of CRC occurrence and progression and to explore more effective interventions to prevent CRC recurrence.

Previous studies have shown that metabolic disorders contribute to the formation of a protumorigenic environment and are associated with the risk of CRC (4). Metabolomics, as a branch of systems biology following genomics, transcriptomics, and proteomics, was established to detect and analyze the profiling of metabolites in biofluids, cells, and tissues (5). Metabolomics is a technology used for identifying endogenous compounds as biomarkers in diseases and for unveiling potential mechanisms associated with disease processes (6). A variety of analytical platforms, including high-performance liquid chromatography/mass spectrometry (HPLC/MS), UPLC time-of-flight MS (UPLC-TOF-MS), and ultra-performance LC–MS (LC–MS), have been used to profile metabolites in various tumors by using tissue, urine, and serum samples (7, 8). A variety of metabolites, including tyrosine and glutamine–leucine, have been identified as biomarkers for the early diagnosis of CRC (9, 10). Our previous study also showed the important predictive role of hexadecanedioic acid, 4-dodecylbenzenesulfonic acid, 2-pyrocatechuic acid, and formylanthranilic acid in CRC patients by comparing serum differential metabolites between CRC patients and healthy controls (11).

Currently, surgical resection, including sufficient adjacent large intestine, mesentery, nearby lymph nodes, and blood vessels, is still the mainstay treatment for CRC patients (12). However, there are few studies exploring the changes in the serum metabolite profiles before and after surgery for patients with CRC. The metabolic impact of radical tumor resection on CRC patients remains unknown. In addition, there is still a lack of effective biomarkers for monitoring tumor recurrence after surgery. In our study, metabolomic technology based on ultra-high-performance liquid chromatography (UHPLC) was used to analyze the metabolic differences in the serum of CRC patients before and after radical surgical resection. The identification of affected metabolites and metabolic pathways may reveal the metabolic alterations associated with radical resection of colorectal tumors, providing insights into the comprehensive physiological changes associated with radical resection for CRC and improving guidelines for postoperative care, which may offer new methods for predicting the prognosis of surgical resection in CRC patients.

## Materials and methods

### Participants

The inclusion criteria for the healthy control group were healthy adults aged 18–80 years without any forms of cancer. Patients were included in the CRC group of this research if they met the following inclusion criteria: those who were 18–80 years old, had a definite diagnosis of carcinoma by biopsy pathology through colonoscopy or proctoscopy, successfully underwent radical resection of CRC, and had provided informed consent to participate in the study.

Patients who met the following conditions were excluded: those who had any other malignancy; had chronic renal failure; had human immunodeficiency virus infection or metabolic diseases, such as diabetes, fatty liver and obesity; or declined to participate.

A total of 50 healthy volunteers and 67 CRC patients were recruited for this study at the Peking Union Medical College Hospital between 2019 and 2021. All 67 CRC patients underwent radical resection of CRC by the same experienced surgical team. Information on the clinical characteristics of the patients, namely, sex, age, body mass index (BMI), level of serum carcinoma embryonic antigen (CEA), and histopathology classification, was collected and analyzed retrospectively. The research conformed to the Declaration of Helsinki and the guidelines of ethical standards. The research was reviewed and approved by the Ethics Committee of Peking Union Medical College Hospital. Signed informed consent was provided by all participants in this research.

### Collection and preparation of samples

All blood samples were collected in the morning after fasting for 12 h, centrifuged at 3,000 rpm for 15 min at 4°C to separate the serum within 2 h, and preserved at −80°C for subsequent analysis. Blood samples of CRC patients were collected just before surgery and 1 week after the operation.

All serum samples were thawed at 4°C for approximately 1 h. Then, a 150 µl of serum was added to a 100 µl of freshly prepared internal standard solution and a 450 µl of precooled formaldehyde. The thoroughly mixed samples in a 2.0-ml Eppendorf tube were then centrifuged at 14,000 × *g* for 15 min at room temperature. The resulting supernatants were collected and dried in a centrifugal vacuum evaporator for 18 h.

### Metabolomic analysis

The identification and quantitation of metabolites were performed by LC–MS/MS analyses using UHPLC on a Thermo Scientific UltiMate Dionex 3000 RSLC HPF system (Thermo Fisher Scientific, U.S.) with a UPLC BEH Amide column (2.1 mm × 100 mm, 1.7 µm) coupled to Q-Exactive Orbitrap (Thermo Fisher Scientific, USA). The instrument operated at a 60,000 resolution with a full mass scan ranging from 67 to 1,000 *m/z*. The mobile phase consisted of 25 mmol/L ammonium acetate plus an aqueous solution of 25 ammonia hydroxide (pH = 9.75) (A), and acetonitrile (B) was used to conduct chromatographic separation at 3 µl/min at 4°C.

Under the control of acquisition software (Xcalibur, Thermo Fisher Scientific), a QE HFX mass spectrometer was used to acquire MS/MS spectra in information-dependent acquisition (IDA) mode. The parameters are detailed as follows: sheath gas flow rate=30 Arb, aux gas flow rate=25 Arb, capillary temperature=350°C, full MS resolution=60,000, MS/MS resolution=7,500, and collision energy=10/30/60 in NCE mode. All the serum samples were complementarily analyzed in positive ion mode (+3.6 kV) and negative ion mode (−3.2 kV) in metabolic profiling studies. The metabolites were identified on the basis of a detected chromatographic peak with an associated retention time and a unique accurate *m/z* for UHPLC. MetaboScape 3.0 software was used for UHPLC data analyses including peak extraction, denoising, and normalization.

### Statistical analysis

SIMCA software (V16.0.2, Sartorius Stedim Data Analytics AB, Umea, Sweden) was employed to perform logarithmic (LOG) and centralization (CTR) conversions on the data. Then, principal component analysis (PCA) and orthogonal projections to latent structures-discriminant analysis (OPLS-DA) were conducted to estimate the classification and validate grouping trends in different groups. A 200-iter permutation test for the OPLS-DA model was implemented to prevent overfitting. The intercept of R2 on the *Y*-axis was less than 0.4 and that of Q2 on the *Y*-axis was less than 0, indicating that the model was not overfitting with good robustness.

A paired *t*-test was conducted to compare the differences in metabolites between the pre- and postoperation groups. Differences in metabolites between CRC patients and healthy volunteers were compared with the unpaired Student's *t*-test. The VIP value represents the overall contribution and importance of each *X*-variable. A VIP value of <1 was considered significant. A *p*-value of <0.05 was considered statistically significant. MetaboAnalyst 4.0 (http://www.metaboanalyst.ca/MetaboAnalyst/) was used for pathway analysis and enrichment analysis based on the Kyoto Encyclopedia of Genes and Genomes (KEGG, http://www.genome.jp/kegg/) database and Small Molecule Pathway Database (SMPDB, http://smpdb.ca/) to further explore the metabolic pathways involved in the change in metabolites. iPath 3.0 (http://pathways.embl.de/) was used for the visualization of the crucial metabolites and significant metabolic pathways.

## Results

### Characteristics of participants

The population demographics and clinical characteristics of our study subjects are presented in Table 1, and these are sex, age, BMI value, level of serum CEA, and cancer location. The mean age was 60.79 years for patients with CRC and 61.52 years for controls (*p* = 0.648). There was also no difference in BMI or sex between CRC patients and healthy controls (*p* = 0.214, 0.88). In all 67 patients, the diagnosis of colon carcinoma was confirmed by a histopathological examination in 32 patients, and rectal carcinoma was confirmed in 35 patients. The mean value of CEA was 5.91 mmol/L for CRC patients.

**Table 1:** Clinical characteristics of the study population.

Parameters	CRC group (*N* = 67)	Control group (*N* = 50)	*p*-Value
Gender			
Male, *n* (%)	42 (62.7%)	32 (64.0%)	
Female, *n* (%)	25 (37.3%)	18 (36.0%)	0.88
Age, mean (range)	60.79 (40–87)	61.52 (50–82)	0.648
BMI (kg/m^2^), mean ± SD	24.39 ± 3.31	25.13 ± 3.01	0.214
CEA (ng/ml), mean ± SD	5.91 ± 10.63	—	
Cancer location			
Colon, *n* (%)	32	—	
Rectum, *n* (%)	35	—	

A value of *p* < 0.05 was considered significant. BMI, body mass index; CEA, carcinoembryonic antigen; SD, standard deviation.

### Metabolic profiling of colorectal cancer patients

**Figure 1:**
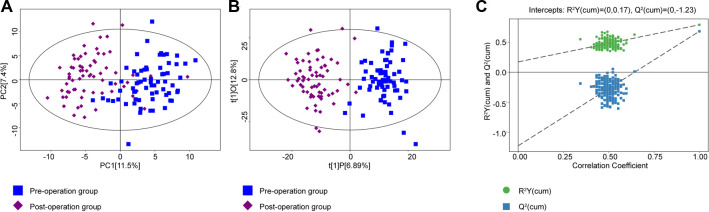
(**A**) PCA was conducted between the preoperation group (blue dots) and postoperation group (purple dots). (**B**) The OPLS-DA model was constructed using data from 67 CRC patients' serum metabonomics before (blue) and after (purple) surgery. (**C**) The permutation test plot of OPLS-DA (green, the value of *R*^2^*Y*; blue, the value of *Q*^2^; permutation test with 200 times, a *p* value of CV-ANOVA < 0.01). PCA, principal component analysis; OPLS-DA, orthogonal projections to latent structures-discriminant analysis; CRC, colorectal cancer.

A total of 180 metabolites were detected by UHPLC. PCA and OPLS-DA were used to perform discriminative pattern recognition analysis for the metabolite data. PCA is an unsupervised projection method that converts a set of observed potentially correlated variables into linearly unrelated variables by orthogonal transformation. In our research, PCA revealed that the metabolomic characteristics between the pre- and postoperation groups were significantly different (Figure 1A). OPLS-DA score plots also displayed a well-distinguished and clustered pattern between pre- and postoperative patients (Figure 1B). The permutation test verified the validity of the OPLS-DA model with all permuted *Q*^2^ and *R*^2^ values lower than the original values, and the *Q*^2^ (cum) intercepted the *Y*-axis at −1.23 (Figure 1C).

### Identification of different metabolites

**Figure 2:**
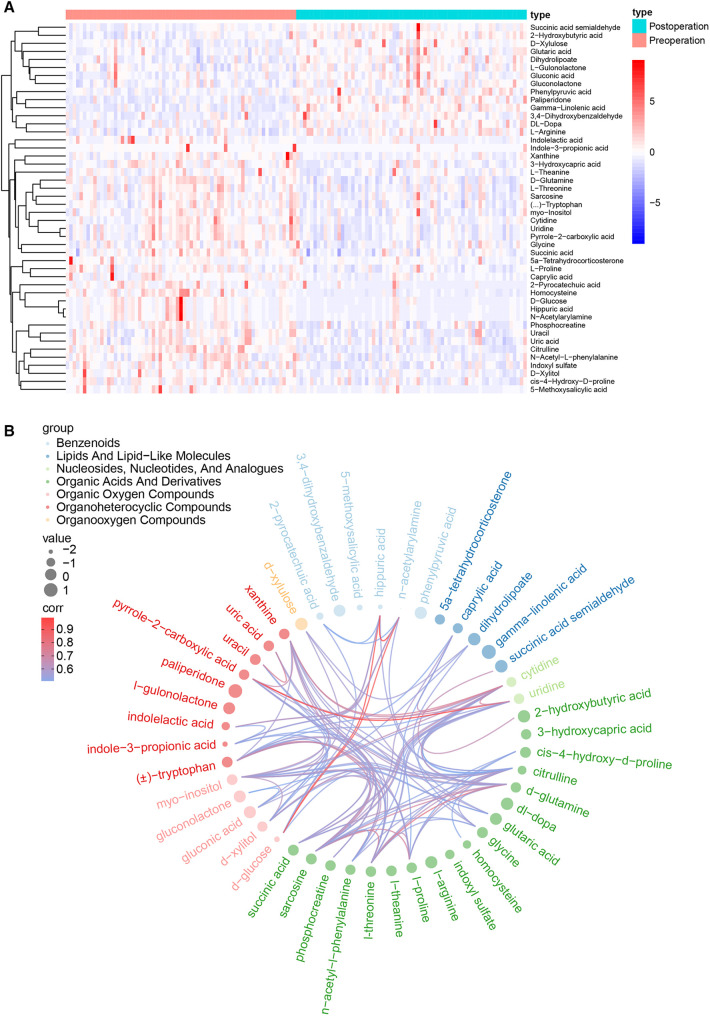
(**A**) The heat map for the 46 critical metabolites between the pre- and postoperation groups, including 32 downregulated metabolites and 14 upregulated metabolites. (**B**) The correlation network of the 46 differentially expressed metabolites.

Based on the variable importance of the projection (VIP) threshold and *p* values in the paired-samples *t*-test for metabolites identified through the OPLS-DA model that we established, 46 differentially expressed metabolites were screened between pre- and postoperation groups with the criteria of VIP > 1 and *p* values <0.05. The log fold change values represent the difference between the two groups. Thirty-two metabolites with log fold change values <0 were downregulated, while 14 metabolites with log fold change values >0 were upregulated in postoperative patients compared with those in preoperative patients. The expression levels of these metabolites are presented in the heat map (Figure 2A). In addition, the correlations of these differentiated metabolites are declared by chord analysis in Figure 2B.

### Metabolic pathway analysis

**Figure 3:**
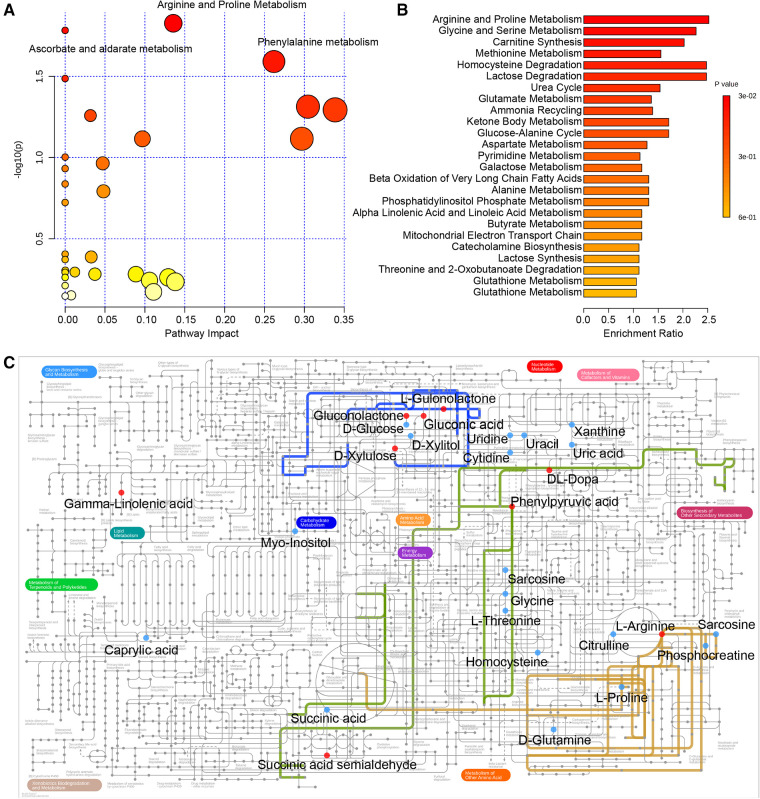
(**A**) Pathway analysis based on the KEGG database. (**B**) Enrichment analysis based on SMPDB. (**C**) Metabolic network of the crucial metabolites and significant metabolic pathways in the KEGG general metabolic pathway map. The following colored dots and lines denote the following: red dots, increased metabolites after surgery; blue dots, decreased metabolites after surgery; blue line, ascorbate and aldarate metabolism; yellow line, arginine and proline metabolism; green line, phenylalanine metabolism. KEGG, Kyoto Encyclopedia of Genes and Genomes; SMPDB, Small Molecule Pathway Database.

Next, pathway analysis was conducted on the basis of the KEGG database for the differentially expressed metabolites. The metabolic pathways potentially associated with these 46 differential metabolites were screened and are presented in Table 2 and Figure 3A, including arginine and proline metabolism, ascorbate and aldarate metabolism, and phenylalanine metabolism (*p *= 0.0149, 0.0165, and 0.0256). In addition, the enrichment analysis module of MetaboAnalyst 4.0 based on the metabolite sets from SMPDB was used to strengthen the understanding of the altered metabolic pathways and validate the correlation of arginine and proline metabolism with radical resection of CRC (Figure 3B, **Supplementary Table S1**, *p *= 0.0272). The metabolic network of the differential metabolites and altered metabolic pathways in the KEGG general metabolic pathway map is shown in Figure 3C.

**Table 2:** Pathway analysis for the 46 critical metabolites based on KEGG.

Pathway name	Total	Hits	*p*	−log(*p*)	Impact
Arginine and proline metabolism	38	4	0.015	1.8266	0.13566
Ascorbate and aldarate metabolism	8	2	0.016	1.7829	0
Phenylalanine metabolism	10	2	0.026	1.591	0.2619
Aminoacyl-tRNA biosynthesis	48	4	0.033	1.4862	0
Arginine biosynthesis	14	2	0.049	1.3134	0.30457
Glycine, serine, and threonine metabolism	33	3	0.051	1.2935	0.3387
Butanoate metabolism	15	2	0.055	1.2583	0.03175
Pentose and glucuronate interconversions	18	2	0.077	1.1158	0.29688
Pyrimidine metabolism	39	3	0.077	1.1154	0.09694
Phenylalanine, tyrosine, and tryptophan biosynthesis	4	1	0.099	1.0027	0
Pentose phosphate pathway	22	2	0.108	0.96468	0.04712

### Identification of the significant metabolites in colorectal cancer

**Figure 4:**
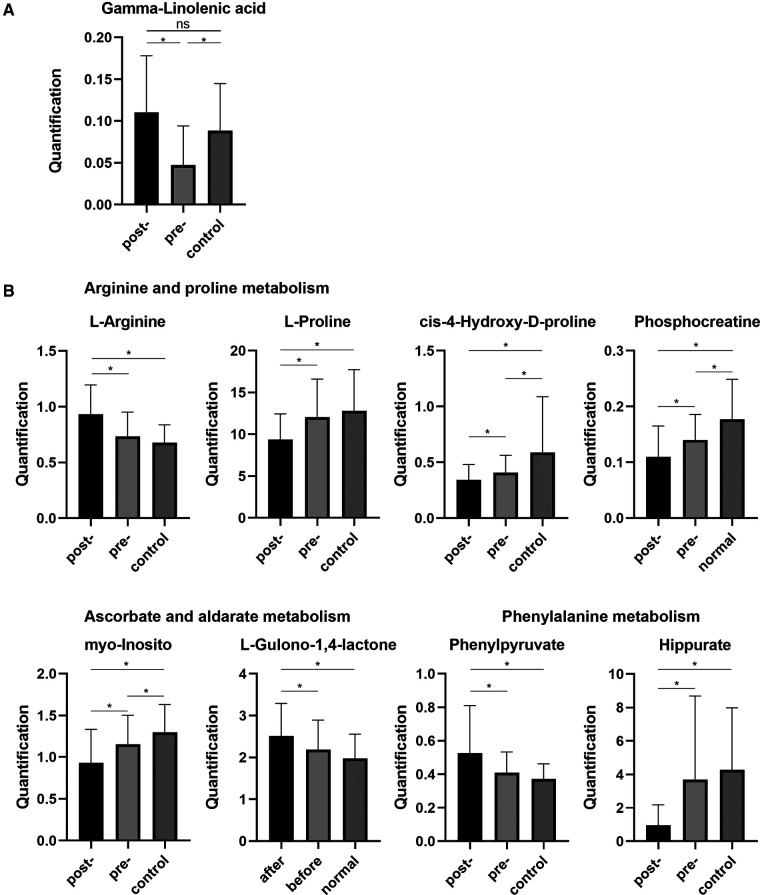
(**A**) Serum levels of GLA in healthy samples and preoperative and postoperative samples. (**B**) The expression levels of metabolites involved in the metabolic pathways associated with radical resection of CRC. GLA, gamma-linolenic acid. CRC, colorectal cancer. *p < 0.05. ns, not significant.

By combining the serum metabolites in the control group, we identified significantly altered metabolites for both the preoperative group vs. the postoperative group and the preoperative group vs. the control group. Interestingly, there was one metabolite, gamma-linolenic acid (GLA), which was evidently decreased in the preoperative CRC patients compared with that in healthy volunteers, and then returned to the healthy group level after the operation (Figure 4). Ultimately, we selectively compared the expression levels of metabolites involved in the metabolic pathways associated with radical resection of CRC. In the process of arginine and proline metabolism, l-arginine in the postoperation group was increased, while L-proline, *cis*-4-hydroxy-d-proline, and phosphocreatine in the postoperation group were decreased compared with those in the healthy control group. The serum levels of *cis*-4-hydroxy-d-proline and phosphocreatine in CRC patients before hydroxy were significantly lower than those in healthy volunteers. In the metabolic pathways of ascorbate and aldarate, the preoperative myo-orthosito was significantly lower than that of healthy volunteers and higher than that of postoperative patients, while the postoperative l-gulono-1,4-lactone was significantly higher than that of healthy volunteers without any difference between the preoperative and healthy groups. Among the phenylalanine metabolism pathways, the postoperative phenylpyruvate was increased, while postoperative hippurate was prominently decreased compared with that of healthy volunteers.

## Discussion

In this study, we used UHPLC to conduct serum untargeted metabolomic analysis for CRC patients and revealed the characteristic differences in the metabolomic profiles in CRC patients before and after surgery. Furthermore, the significant metabolic pathways related to CRC were also identified.

Our results showed that the metabolic profiles of arginine and proline were significantly altered in the serum of patients before and after surgery, among which, l-proline, *cis*-4-hydroxy-d-proline, and *cis*-4-hydroxy-d-proline were significantly downregulated after surgery. However, l-arginine was upregulated after surgery. As a nonessential amino acid, proline is the only proteinogenic secondary amino acid with an *α* amino group within the pyrrolidine ring (13). Proline is an important product of glutamine, which can be converted to proline *via* pyrroline-5-carboxylate/glutamate-γ-semialdehyde (P5C/GSA) (14). Proline and P5C are interconvertibly catalyzed by proline dehydrogenase/proline oxidase (PRODH/POX) and PYCRs, which play a role in maintaining redox homeostasis between the cytosol and the mitochondria, and contribute to the recycling of cellular NAD(P)H to NAD(P). Recently, studies have focused on the conducive role of proline for metabolic reprogramming of cancer and its clinical significance (14–16). Two well-recognized oncogenes, namely, c-MYC and phosphoinositide 3-kinase (PI3K), robustly upregulate the proline biosynthetic pathway by increasing its enzymes (P5CS, PYCR1/2/L) (13). Meanwhile, proline catabolism involving PRODH/POX, which is regulated by P53, peroxisome proliferator-activated receptor γ (PPARγ), and AMP-activated protein kinase (AMPK), can also initiate ROS-mediated senescence, apoptosis, and autophagy in tumors. The process of proline synthesis has been demonstrated to be necessary for tumor cell growth, and an increased level of proline synthesis has been observed in breast metastatic cancer cells, melanoma cells, and ovarian cancer stem cells (14). In addition, in lung cancer cell lines expressing high levels of endogenous MYC and proline synthetic enzymes, a knockdown of proline enzymes or MYC could significantly inhibit tumor growth. Supplementation with proline or P5C partly rescued the decrease in proliferation induced by a knockdown of P5CS or PYCR1/2. A high expression of PYCR1 was also significantly associated with poor prognostic molecular subtypes of breast cancer, which is consistent with our results of downregulated proline levels post operation compared with that of the preoperative period. However, its underlying mechanisms remain unclear. Here, it deserves mention that, not necessarily due to the disruption of protein synthesis, the proline metabolic pathway also functions to regulate redox-dependent signaling through parametabolic mechanisms. In tumors, proline for protein synthesis is lower than that in adjacent normal tissues. On the other hand, proline is also indispensable for tumors to help manage stress during tumorigenic growth (13). This might explain why there were no differences in proline levels in our results between samples from preoperative patients and healthy people.

It is generally accepted that ascorbate has a potential therapeutic effect on tumors by generating sustainable ascorbate radical and H_2_O_2_ in extracellular fluid (17). However, the underlying mechanisms for the selective cytotoxicity to cancer cells but not to normal ascorbate cells are not well understood. Recent research showed that high-dose intravenous ascorbate as an adjuvant could improve chemosensitivity and relieve the toxicity of chemotherapy for ovarian cancer, which was related to an activation of the ATM/AMPK pathway and inhibition of mTOR signaling due to DNA damage and ATP depletion triggered by ascorbate-generated ROS (18). In addition, another study reported that some genes involved in ascorbate and aldarate metabolism were overexpressed in adenocarcinoma compared with those in normal tissues (19). It has also been reported that ascorbate and aldarate metabolism are significantly disordered in renal cell carcinoma (20). In CRC, intravenous ascorbate is associated with the inhibition of tumor glycolysis, thus advancing tumor regression and improving the tolerance and side effects of chemotherapy (21, 22). However, the impact of surgical resection of CRC tumors on ascorbate and aldarate metabolism has not been studied. We found that the levels of compounds involved in ascorbate and aldarate metabolism increased following surgery, which may play a potential role in inhibiting tumor recurrence and may be a potential factor that improves the prognosis of CRC.

We also noticed that phenylalanine metabolism changed after the operation. Previous studies have shown the significant differences in serum phenylalanine levels between gastroesophageal cancer patients and healthy volunteers, which may be accounted for by the dysfunction of phenylalanine hydroxylase activity in inflammatory or malignant disease states (23–25). In some tumors such as melanoma, hepatocarcinoma, leukemia, and breast cancer, lowering the serum levels of tyrosine and phenylalanine could inhibit the growth and metastases of tumors and improve the response to chemotherapy. A low tyrosine and phenylalanine diet might be correlated with the stabilization and regression of cancer (26). However, the underlying mechanism remains unclear. In our research, instead of serum levels of phenylalanine or tyrosine, significant changes in phenethylamine and hippurate levels were observed compared with those before surgery. Even so, we think the elevated phenylpyruvate levels can also indicate the reduced activity of phenylalanine hydroxylase in patients with CRC, which may suggest that it is necessary to control and monitor serum phenylalanine levels after surgery.

GLA, as a polyunsaturated fatty acid (PUFA), is an intermediate product in the process of linoleic acid (all *cis*-6,9-octadecadienoic acid) metabolism (27). Recently, GLA has gained importance due to its antifibrotic, anti-inflammatory, and tumoricidal properties with little or no side effects. Previous studies have reported that low GLA levels are associated with the progression of inflammatory breast cancer (28). In mammary gland carcinoma induced by 7,12-dimethylbenz (*a*) anthracene (DMBA), GLA has been proven to activate the mitochondrial-mediated death apoptosis pathway, regulate hypoxia-induced cell signaling, and decrease the synthesis of *de novo* fatty acids fatty acid to execute its antitumor effect by mediating the cholinergic anti-inflammatory pathway (29). In addition, GLA has been reported to decrease the expression of the metastasis-associated protein osteonectin (or SPARC) and increase the expression of metastasis suppressor genes to inhibit tumor metastasis. GLA could also decrease the β-catenin expression of Wnt/b-catenin signaling pathway to inhibit gastric cancer cell growth and EMT induced by hypoxia (30). Additionally, polyunsaturated fatty acids, including GLA, could induce an apoptosis of colon cancer cells (31). However, further research is needed to determine the impact of GLA on CRC and its underlying mechanism. In our results, the preoperative serum GLA levels of CRC patients were significantly lower than those of the normal control group, which is consistent with the tendency described in previous studies. Interestingly, we observed that postoperative serum GLA levels in CRC patients were significantly elevated and approached the preoperative level, highlighting the potential role of GLA as an effective biomarker for CRC to help predict prognosis following surgery and detect postoperative recurrence.

Although we found that CRC surgery resection could bring about changes in some metabolic pathways, including arginine and proline metabolism, phenylalanine metabolism, and ascorbate and aldarate metabolism, there is no evidence in serum metabolomics that surgery can reverse the metabolic reprogramming caused by CRC. In addition, limited by the sample size, we could not group tumors according to their clinical stage, which might have effects on serum metabolites. In future studies, larger sample sizes and longer follow-up periods are necessary to explore the metabolomic profile associated with CRC and confirm its prognostic value. Furthermore, metabolomic information from CRC patients with adjuvant chemotherapy is needed to analyze differences in the sensitivity of different metabolomic characteristics to chemotherapy and the target metabolites for adjuvant treatments.

## Conclusion

In summary, our study established a serum metabolomic signature associated with CRC surgery resection and explored the physiological alterations in CRC patients after surgery. The results suggested that removal of the tumor may affect the metabolic profiling of organisms caused by CRC, which provides insights into the scheme for postoperative nutritional support and postoperative adjuvant therapy. In addition, we identified the diagnostic and prognostic value of GLA as a novel CRC effective biomarker that can monitor treatment efficacy and tumor recurrence.

## Data Availability

The raw data supporting the conclusions of this article will be made available by the authors without undue reservation.
