# Supplementary material for: Metabolomic profiling identifies biomarkers and metabolic impacts of surgery for colorectal cancer
Source: Front Surg. 2022 Aug 25;9:913967. doi: 10.3389/fsurg.2022.913967 (PMC9453208; doi:10.3389/fsurg.2022.913967)
Supplement: Supplementary file 1 [file Table_1_v1.docx]

| Supplementary Table 1. Results of Enrichment Analysis based on SMPDB | | | |
| --- | --- | --- | --- |
| Metabolite Set | Total | Hits | P value |
| Arginine and Proline Metabolism | 53 | 6 | 0.0272 |
| Glycine and Serine Metabolism | 59 | 6 | 0.0435 |
| Carnitine Synthesis | 22 | 2 | 0.26 |
| Methionine Metabolism | 43 | 3 | 0.304 |
| Homocysteine Degradation | 9 | 1 | 0.34 |
| Lactose Degradation | 9 | 1 | 0.34 |
| Urea Cycle | 29 | 2 | 0.378 |
| Glutamate Metabolism | 49 | 3 | 0.38 |
| Ammonia Recycling | 32 | 2 | 0.427 |
| Ketone Body Metabolism | 13 | 1 | 0.452 |
